# Evidence for altered upper extremity muscle synergies in chronic stroke survivors with mild and moderate impairment

**DOI:** 10.3389/fnhum.2015.00006

**Published:** 2015-02-11

**Authors:** Jinsook Roh, William Z. Rymer, Randall F. Beer

**Affiliations:** ^1^Department of Kinesiology, Temple UniversityPhiladelphia, PA, USA; ^2^Department of Physical Medicine and Rehabilitation, Feinberg School of Medicine, Northwestern UniversityChicago, IL, USA; ^3^Sensory Motor Performance Program, Rehabilitation Institute of ChicagoChicago, IL, USA; ^4^Department of Biomedical Engineering, Northwestern UniversityChicago, IL, USA

**Keywords:** motor control, muscle synergy, stroke, electromyography, neurorehabilitation

## Abstract

Previous studies indicate that motor coordination may be achieved by assembling task-dependent combinations of a few muscle synergies, defined here as fixed patterns of activation across a set of muscles. Our recent study of severely impaired chronic stroke survivors showed that some muscle synergies underlying isometric force generation at the hand are altered in the affected arm. However, whether similar alterations are evident in stroke survivors with lesser impairment remains unclear. Accordingly, we examined muscle synergies underlying spatial patterns of elbow and shoulder muscle activation recorded during an isometric force target matching protocol performed by 16 chronic stroke survivors, evenly divided across mild and moderate impairment levels. We applied non-negative matrix factorization to identify the muscle synergies and compared their structure across groups, including previously collected data from six age-matched control subjects and eight severely impaired stroke survivors. For all groups, EMG spatial patterns were well explained by task-dependent combinations of only a few (typically 4) muscle synergies. Broadly speaking, elbow-related synergies were conserved across stroke survivors, regardless of impairment level. In contrast, the shoulder-related synergies of some stroke survivors with mild and moderate impairment differed from controls, in a manner similar to severely impaired subjects. Cluster analysis of pooled synergies for the 30 subjects identified seven distinct clusters (synergies). Subsequent analysis confirmed that the incidences of three elbow-related synergies were independent of impairment level, while the incidences of four shoulder-related synergies were systematically correlated with impairment level. Overall, our results suggest that alterations in the shoulder muscle synergies underlying isometric force generation appear prominently in mild and moderate stroke, as in most cases of severe stroke, in an impairment level-dependent manner.

## Introduction

Broadly speaking, disturbances of motor function following stroke can be attributed to three primary impairments: reduced corticospinal drive to agonist muscles (weakness), altered reflex activity (e.g., spasticity), and impaired motor coordination (Twitchell, 1951; Brunnstrom, 1970; Knutsson and Mårtensson, 1980). In many patients, when weakness and spasticity are treated effectively, or resolve spontaneously, motor dysfunction remains severe (Landau, 1980; Hesse et al., 1996; Dewald et al., 2001). Accordingly, understanding the mechanisms that underlie impaired motor coordination following stroke is essential for the design of effective rehabilitation protocols.

Behavioral and stimulation-based studies in motor systems suggest that normal neuromuscular coordination can be achieved by activating a relatively limited number of muscle synergies, each of which represents a pattern of muscle activation with distinct spatial (and in some formulations, temporal) characteristics (Tresch et al., 1999; Ivanenko et al., 2004; Cheung et al., 2005; d’Avella and Bizzi, 2005; d’Avella et al., 2006, 2008; Torres-Oviedo et al., 2006; Torres-Oviedo and Ting, 2007; Kargo et al., 2010; Overduin et al., 2012). Muscle synergies have been identified as building blocks for a variety of motor tasks in humans, including postural responses (Krishnamoorthy et al., 2003; Weiss and Flanders, 2004; Torres-Oviedo and Ting, 2007), locomotion (Clark et al., 2010; Monaco et al., 2010), hand shaping and signing (Santello et al., 1998; Weiss and Flanders, 2004; Ajiboye and Weir, 2009), isometric force generation in the upper extremity (Roh et al., 2012), and reaching movements performed under different biomechanical constraints (Sabatini, 2002; d’Avella et al., 2006, 2008; Cheung et al., 2009a; Muceli et al., 2010). Furthermore, muscle synergies generalize across different task constraints, as shown in recent animal studies of postural responses involving different perturbation types and postures (Torres-Oviedo et al., 2006) as well as voluntary motor behaviors (Roh et al., 2011). Similarly, muscle synergies have been shown to be quite robust in intact humans (Valero-Cuevas, 2000; Ivanenko et al., 2004; d’Avella et al., 2006, 2008; Chvatal et al., 2011; Hug et al., 2011; Roh et al., 2012). The use of muscle synergies may facilitate control of task-level variables (Ting and Macpherson, 2005; Torres-Oviedo et al., 2006; McKay and Ting, 2008), and/or simplify the generation of motor behaviors by reducing the dimensionality of the control problem associated with mechanically redundant musculature (Grillner, 1985; Bizzi et al., 1991; Tresch et al., 1999; Fetz et al., 2000; Tresch et al., 2002; Miller, 2004; Bizzi et al., 2008; d’Avella and Lacquaniti, 2013).

Several recent studies have examined how stroke impacts the modular control of voluntary limb movements (Cheung et al., 2009b, 2012; Clark et al., 2010).The initial study, which focused on reaching movements performed by a group of chronic stroke survivors with predominantly mild impairment, concluded that stroke altered the recruitment patterns of normal muscle synergies, rather than altering synergy internal structure (Cheung et al., 2009b). A subsequent study involving subjects with a diverse range of impairment levels confirmed preservation of normal muscle synergies in mildly impaired stroke survivors, but reported evidence of merging and fractionation of normal synergies in more impaired stroke subjects (Cheung et al., 2012). Similarly, Clark et al. (2010) found that fewer muscle synergies were required to reconstruct locomotor muscle activation patterns in more impaired stroke survivors, reflecting an apparent merging of synergies identified in healthy subjects. Overall, these studies suggest that alterations in muscle synergy structure are mainly evident in stroke survivors with severe impairment.

Muscle synergies are potentially shaped by biomechanical or task constraints, independent of putative neural constraints (Todorov, 2004; Kutch and Valero-Cuevas, 2012). Thus, differences in task performance between stroke survivors and healthy controls are a potential confounding factor when comparing the number and structure of muscle synergies underlying voluntary limb movements. Accordingly, we developed an isometric protocol that provides an opportunity to more closely match task variables (i.e., limb posture and required force level) across healthy and impaired individuals. Our initial study, confined to chronic stroke survivors with severe motor impairment, examined muscle synergies underlying isometric force generation at the hand (Roh et al., 2013). In contrast to the results for dynamic tasks (Cheung et al., 2009b, 2012; Clark et al., 2010), we found preservation of the number of muscle synergies in the affected arm of severely impaired stroke survivors and relatively stereotyped alterations in specific muscle synergies related to the activation of shoulder muscles.

As an extension of our previous study (Roh et al., 2013), the current study focused on higher functioning stroke survivors. Specifically, we hypothesized that alterations in muscle synergy structure are also evident in stroke survivors with mild or moderate impairment. To evaluate this hypothesis, subjects with mild and moderate motor impairment completed a 3-D isometric force target matching protocol identical to Roh et al. (2013). Synergies underlying shoulder and elbow muscle activations were identified using non-negative matrix factorization, and compared with those previously identified in age-matched healthy subjects and severely impaired stroke survivors.

## Materials and methods

### Participants

We recruited eight stroke survivors with mild (Fugl-Meyer (FM) score >50) and moderate (FM between 26 and 50) impairment, respectively. Additionally, previously collected data from six age-matched controls and eight severely impaired (FM < 26) stroke survivors (Roh et al., 2013) were re-analyzed and incorporated in this study. The datasets of the stroke survivors with severe impairment (*n* = 8) were a randomly selected subset of datasets (*n* = 10) published in our previous work; this random selection matched the number of datasets for the stroke groups with mild and moderate impairment (*n* = 8, respectively). Other randomly chosen subsets from the severely impaired group provided analytic results consistent with those presented in Results. Demographic and clinical data for the stroke survivors and control subjects are summarized in Table 1. All control subjects and 22 out of 24 stroke survivors self-reported as right-hand dominant. The study was performed in accordance with the Declaration of Helsinki, with the approval of the Northwestern University Institutional Review Board. Informed consent was obtained from each subject prior to testing.

**Table 1:** **Participant demographics**.

		Mean	SD	Range
Hemiparetic group (*n* = 24)
Mildly impaired (*n* = 8)
Age (yr)		55.6	9.5	46–70
Months since stroke		51.0	24.6	23–89
Fugl-Meyer score (/66)		55.3	5.3	50–66
Fugl-Meyer score (/22)		19.6	2.3	16–22
Modified Ashworth score (FL/EX)		0.3/0.3	0.6/0.5	0–1+/0–1
Sex (M/F)	5/3
Side affected (L/R)	2/6
Moderately impaired (*n* = 8)
Age (yr)		56.0	8.7	44–68
Months since stroke		82.1	60.0	21–179
Fugl-Meyer score (/66)		36.1	7.0	29–45
Fugl-Meyer score (/22)		16.1	2.2	13–19
Modified Ashworth score (FL/EX)		1.4/0.8	0.7/0.7	0–2/0–2
Sex (M/F)	6/2
Side affected (L/R)	4/4			
Severely impaired (*n* = 8)
Age (yr)		61.8	10.0	53–81
Months since stroke		174.8	94.7	68–302
Fugl-Meyer score (/66)		17.5	3.8	12–23
Fugl-Meyer score (/22)		10.3	1.6	8–12
Modified Ashworth score (FL/EX)		2.4/0.8	1.0/0.8	1+–4/0–2
Sex (M/F)	3/5
Side affected (L/R)	3/5
Control group (*n* = 6)
Age		63.2	7.6	52–73
Sex (M/F)	4/2

*Fugl-Meyer scale: maximum score for entire assessment (/66) and subscore for components related to shoulder and elbow function, excluding pronation/supination (/22). Modified Ashworth score for the elbow: 0 = normal function; 4 = severe spasticity; 1+ was assigned a value of 1.5 to calculate the mean and SD. M, male; F, female. FL, flexion; EX, extension. R, right; L, left*.

### Equipment

Hand position and 3-D forces generated at the hand were recorded using the Multi-Axis Cartesian-based Arm Rehabilitation Machine (MACARM). The MACARM is comprised of a cubic array of 8 actuators connected, via cables, to a centrally located end-effector (for details see Mayhew et al., 2005; Beer et al., 2008). The MACARM supported the subject’s arm against gravity so that the targeted magnitude of the actively generated force was uniform across force directions during the experiment. Additionally, a three DOF sensor (Xsens Technologies BV, The Netherlands), strapped to the upper arm, measured rotation of the limb away from the parasagittal plane. Arm rotational angle, forces and handle position were sampled at 64 (*n* = 18 subjects) or 32 Hz (*n* = 12 subjects) and stored on a computer for subsequent analysis. The sign of the force component in the X-direction (medial-lateral) was reversed for the left arm to facilitate subsequent comparisons.

### Electromyography

Surface EMGs were recorded using the Bagnoli-8 EMG system (Delsys Incorporated, Boston, MA). Eight elbow and shoulder muscles were examined: brachioradialis (BRD); biceps brachii (BI); triceps brachii, long and lateral heads (TRI_long_ and TRI_lat_, respectively); deltoid, anterior, medial and posterior fibers (AD, MD, and PD, respectively); and pectoralis major (clavicular fibers; PECT_clav_). Electrodes were placed in accordance with Hermens et al. (1999) and Perotto et al. (1980). EMG signals were amplified (x 1000), band-pass filtered (20–450 Hz) and sampled at 1820 Hz.

### Protocol

**Figure 1:**
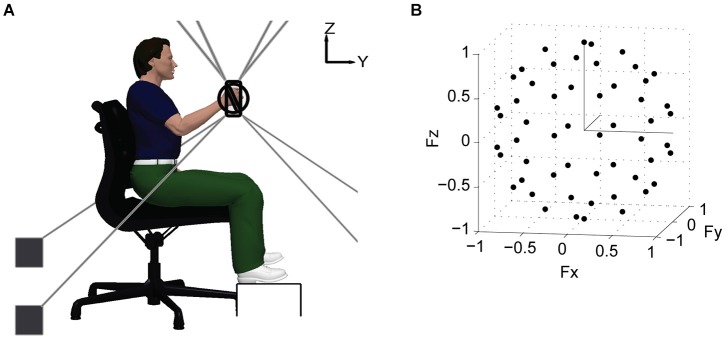
**Schematic of experimental setup and target force directions**. **(A)** lateral view of the experimental setup. Hand position and 3-D forces generated at the hand were recorded using the MACARM cable–robot. The MACARM is comprised of a spatial array of motors (indicated by black filled squares) connected to a central end-effector via cables (depicted by gray lines). The end-effector incorporates a gimbaled handle mounted on a 6-degree of freedom load cell. The coordinate system for position and force measurements is right-handed (i.e., *x* axis is out of the page) and indicated in the right upper corner of the figure. **(B)** the 54 target force directions (black circles) in 3-D force space, represented in Cartesian coordinates.

The general experimental protocol was the same as introduced in Roh et al. (2013). Subjects grasped the MACARM’s gimbaled handle while seated comfortably in an adjustable chair with their hand positioned directly in front of the ipsilateral shoulder at a distance of 60% of arm length (Figure 1A). Wrist and trunk movements were restrained with a commercially available brace and strapping, respectively. Additional strapping was used for some stroke survivors to prevent slippage of the hand from the handle during force generation. Subjects were instructed to maintain their limb in the parasagittal plane during target matching. Changes in shoulder position and arm rotational angle were monitored and verbally corrected if needed, as described in Roh et al. (2013). Prior to data collection, MACARM supported the subject’s relaxed arm against gravity and the load cell was re-zeroed. This procedure ensured that the targeted magnitude of the actively generated force was uniform across force directions.

Following a short training session, subjects generated voluntary forces in 54 different directions (black dots; Figure 1B), approximately uniformly distributed in 3-D force space. Force magnitude was set at 40% of maximum lateral force, the weakest direction for all subjects. For each trial, a 2-s baseline interval was followed by the time allowed to achieve a target match (7 and 9 s for control and stroke subjects, respectively). A successful target match required the subject to maintain the center of a force-driven cursor within a target sphere for 0.8 s. Subjects were given 3 attempts to match each target, before proceeding to the next target in the random sequence. If there were unmatched target directions after the initial full set of trials, they were repeated without a constraint on limb rotation. Subjects were instructed to allow the MACARM to support the weight of their arm between trials. To minimize the potential for fatigue, an inter-trial interval of 10-s and a 1-min rest after each 10 trial block were provided.

Stroke survivors completed the protocol with their contralesional limb only (a mix of dominant and non-dominant limbs). We used data recorded from age-matched neurologically intact subjects to characterize normative performance, since a number of studies have reported motor deficits in the ipsilesional limb (Yarosh et al., 2004; Mirbagheri et al., 2007). Healthy participants completed the protocol with both limbs, tested in random order in separate sessions spaced a few days apart.

### Data analysis

Data analysis was performed using Matlab (The MathWorks, Inc.). EMGs were demeaned, rectified, and averaged over the 0.8 s target matching interval. Mean baseline EMGs for each trial were subtracted from the averaged data for the same trial. Hence, the EMG data for each trial, a vector whose dimension was 8 (the number of muscles recorded), corresponded to active force generation beyond any residual baseline force level. Subsequent analysis confirmed that mean baseline x, y, and z force components were less than 1 N for each group. Prior to synergy identification, EMG data recorded from each muscle were concatenated across trials and normalized to have unit-variance, which prevented bias towards high-variance muscles (Roh et al., 2012).

We applied a NMF algorithm (Lee and Seung, 1999, 2001) to an EMG dataset to identify muscle synergies and their activation weights. An EMG pattern recorded under isometric conditions (*EMG*_isometric_) was modeled as a linear combination of a set of *N* muscle synergies (*W*_isometric_), each of which specified the relative level of activation across 8 muscles (Hart and Giszter, 2004; Cheung et al., 2005; Torres-Oviedo et al., 2006; Tresch et al., 2006; Perreault et al., 2008; Roh et al., 2011, 2012):

(1) $$EMG_{isometric}=W_{isometric}\cdot \;C_{isometric}$$

where *W*_isometric_ was an 8 by *N* matrix containing the *N* synergies (of unit magnitude) in each column and *C*_isometric_ was a *N* by *T* (number of trials) matrix, with each column containing the synergy activation coefficients for a specific trial. For each arm, *EMG*_isometric_ was an 8 by *T* matrix, where *T* was 54 for the control group and ranged from 34–54 for stroke survivors (53–54, 43–54, and 34–54 for groups with mild, moderate, and severe impairment, respectively).

To objectively determine the minimum number of muscle synergies required to reconstruct each data set, we used the higher of the number obtained based on two methods: (1) mean squared error (MSE; Cheung et al., 2005); and (2) a set of criteria based on variance accounted for (VAF; Zar, 1999; Clark et al., 2010; Roh et al., 2013). For the first method, we plotted VAF against the number of synergies and identified the number of muscle synergies at which the VAF curve changed sharply (i.e., the first point on the VAF curve for which the corresponding MSE fell below 5 × 10^−5^). The chosen number indicates that any additional synergies beyond that number capture only a small additional fraction of data variation or noise (Cheung et al., 2005). For the second method, we identified the minimum number of synergies that achieved a mean (across trials) global VAF > 90%, with less than a 3% increase in mean global VAF upon addition of another synergy. As local criteria, the mean VAF for each muscle (muscle VAF) was required to exceed 80%. This procedure ensured that the estimated number of synergies could predict both the overall EMG pattern and the nuances of each dataset. Subsequently, analysis was performed with the typical number (4) of synergies per subject to facilitate comparisons across groups.

We quantified the similarity between the synergies underlying two datasets using the following metrics: the scalar product (*r*-value), global VAF, and muscle VAF (Cheung et al., 2005; Torres-Oviedo and Ting, 2010; Roh et al., 2011, 2012). While the synergy similarity measure (the scalar product; *r*-value) is based on direct comparison of individual synergies, the other metrics are more holistic measures of similarity because they consider the synergy set as a whole. To calculate the synergy similarity measure, individual synergies for two datasets were directly compared by matching them to provide the highest total sum of scalar products. To quantify the similarity of synergies as sets, we calculated the global and muscle VAFs obtained by cross-fitting muscle synergies (i.e., fitting the synergies extracted from dataset *A* to EMG dataset *B*). Readers are referred to Roh et al. (2013) for details of the procedures used to evaluate the statistical significance of *r*-values and the global and muscle VAF measures.

Mean synergies for each group were generated by selecting one set of four synergies to which the synergies from the remaining datasets were matched as described above and then group-averaged. Subsequently, we confirmed that the group-averaged synergies were not sensitive to the choice of initial dataset. To define a normative synergy template, we initially used the mean synergies for the control group and tested whether each of the individual control subject synergies was similar to the associated mean synergy. If any synergy was not similar to the mean, that subject’s synergy set was excluded and we re-calculated the means, until a normative template was obtained for which all of the included synergies were similar. A similar procedure was used to define a synergy template for the severely impaired stroke group. This resulted in exclusion of data for one subject in each group. Subsequently, to examine whether individual muscle synergies were altered post-stroke, we calculated the scalar products (r_norm) of the normative synergies with the corresponding synergies for each dataset (including individual control datasets). Similarly, to examine whether altered synergies identified from the severely impaired stroke group also existed in mildly and moderately impaired stroke survivors, we calculated the scalar products of the severely impaired synergy template with the corresponding synergies for each subject’s data (r_severe). Single factor ANOVA (Matlab) was used to evaluate group differences in *r*-values for each synergy. Additionally, multiple regression (SPSS 22) was used to evaluate relationships between r_norm and clinical characteristics of the stroke survivors (FM subscore, Modified Ashworth score for elbow flexion, and time after stroke-onset). Interaction terms were not considered in the model. The variance inflation factor (VIF) was used to quantify the impact of multicolinearity on the regression results. A VIF < 5 was considered acceptable (Rogerson, 2001).

*Clustering synergies for different subjects*. We pooled the synergies for all subjects and grouped them using hierarchical cluster analysis. Euclidian distance was used as the similarity measure. Accordingly, the procedure was performed by applying the Matlab statistics-toolbox functions *pdist* (Minkowski distance option; *p* = 2), *linkage* (ward option), and *cluster* to the pooled synergy matrix. The number of clusters was determined as the minimum number of clusters partitioning the synergies such that there was not more than one synergy in each cluster from a given subject (Cheung et al., 2005; d’Avella et al., 2006). Subsequently, we used Fisher’s exact test (SPSS 22, IBM, Inc.) to evaluate group differences in the incidence of each cluster.

All statistical tests were made at alpha = 0.05.

## Results

The main aims of this study were to identify muscle synergies underlying isometric force generation in stroke survivors with mild and moderate impairment and to compare them with muscle synergies previously identified in age-matched control and severely impaired subjects (Roh et al., 2013). The targeted force magnitude of the task was 28.4 ± 5.9 N in the control group and 28.4 ± 14.7, 20.6 ± 5.9, and 12.7 ± 5.9 N in the stroke groups with mild, moderate, and severe impairment, respectively. We applied NMF to identify muscle synergies underlying the EMG patterns, which were compared within and across groups. Finally, we grouped the muscle synergies of participants in the control and stroke groups to evaluate whether alterations in synergy patterns reported in severely impaired stroke survivors (Roh et al., 2013) were also observed in the stroke groups with mild or moderate impairment.

### EMG spatial patterns

**Figure 2:**
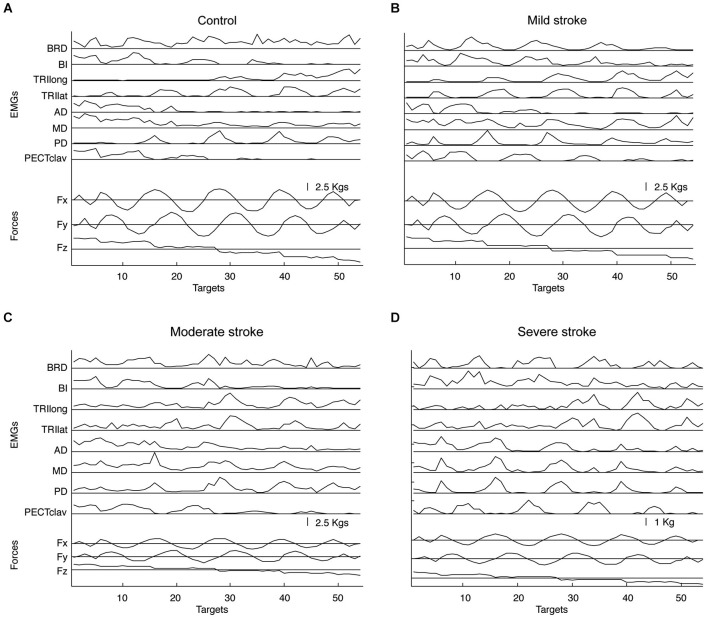
**Representative elbow and shoulder muscle activations across 54 target force directions for age-matched control (A) and stroke subjects with mild, moderate, and severe impairment (B–D), respectively**. Muscle names are abbreviated (BRD, brachioradialis; BI, biceps brachii; TRI_long_ and TRI_lat_, long and lateral heads of triceps brachii, respectively; AD, MD, and PD, anterior, medial and posterior fibers of deltoid, respectively; and PECT_clav_, clavicular fibers of pectoralis major). Lower traces show the force components associated with each target number.

Figure 2 summarizes EMG spatial patterns for a representative subject in each group. As illustrated by Figure 2, subjects in all groups retained the ability to modulate muscle activation with force direction (generated force components are provided at the bottom of each panel). The spatial patterns of EMGs recorded from elbow and shoulder muscles of stroke survivors with mild impairment were qualitatively similar to those of neurologically intact, age-matched subjects as reported in the previous study (Roh et al., 2013). Figure 2 shows that a distinct spatial pattern of activation was recorded from each of the eight muscles. For example, activation of BRD and BI was mainly for force generation in backward (−Fy) and upward (+Fz) directions, while TRI_long_ and TRI_lat_ were activated in response to the required forward (+Fy) and downward (−Fz) force components. At the shoulder, the three heads of deltoid were differentially tuned, with AD primarily activated for medial (−Fx) and upward (+Fz), MD for upward (+Fz) and lateral (+Fx), and PD for lateral (+Fx) and downward (−Fz) forces, respectively. In addition, PECT_clav_ activation was modulated primarily in response to the medial (−Fx) and upward (+Fz) components of the target force.

The most prominent difference in muscle activation patterns between control and higher functioning stroke subjects was observed in the pattern of deltoid activation. While AD and MD, but not PD, tended to be activated together in age-matched control subjects, the activations of all three heads of deltoid appeared more highly correlated in stroke survivors with moderate impairment (see Figure 2C), though not to the extent reported previously for severely impaired stroke survivors (Figure 2D). To identify underlying intermuscular coordination patterns in stroke survivors with mild and moderate impairment, we subsequently performed synergy identification for each EMG dataset.

### Muscle synergies underlying force generation in mildly, moderately, and severely impaired stroke subjects

**Figure 3:**
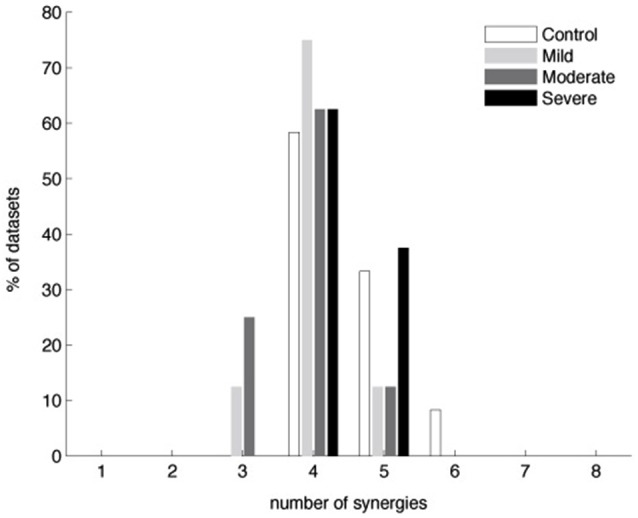
**Number of muscle synergies required to reconstruct muscle activation patterns underlying 3-D isometric force generation in the control and three stroke groups (*n* = 12 and 8 datasets for the control and each stroke group, respectively)**. Similar numbers of synergies were identified by the MSE method as well (see Section Materials and Methods).

**Figure 4:**
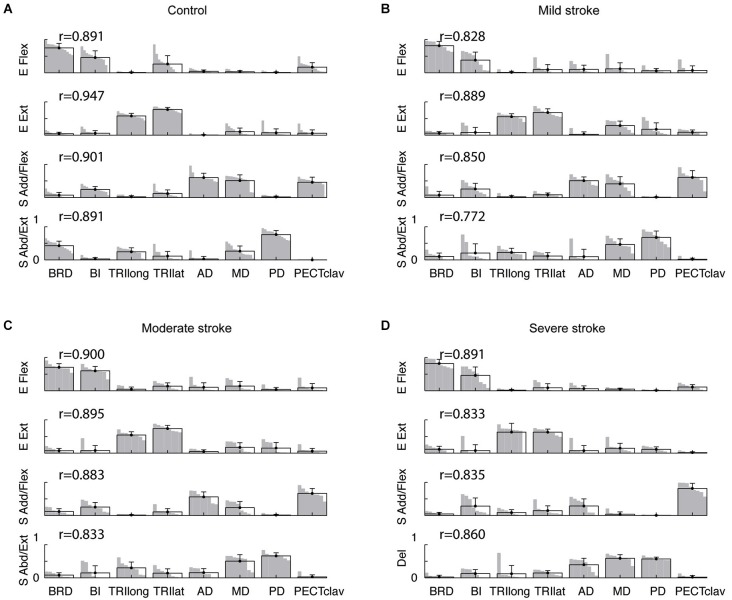
**Composition of muscle synergies for control and stroke groups**. **(A)** in controls, four synergies were identified for the right and left arms of each subject and labeled according to the mechanical action of the main muscles activated within each synergy—elbow flexor (E Flex), elbow extensor (E Ext), shoulder adductor/flexor (S Add/Flex), and shoulder abductor/extensor (S Abd/Ext). **(B–D)** in stroke survivors (mild, moderate, and severe impairment), the structure of the first two synergies (E Flex and E Ext) was relatively similar to that observed in controls. However, changes in the structure of the shoulder synergies were evident in stroke survivors, particularly for the severely impaired group. For each synergy, the group mean muscle weights and standard deviations are superimposed on the distribution of the corresponding muscle weights. Note that the standard deviation of each muscle’s activation is small, indicating that the synergy structure was relatively consistent across subjects within each group. The *r*-values next to each synergy indicate the group-averaged scalar products; the group-mean scalar products were statistically significant (*t*-test, *p* < 0.05), indicating that the synergy structure was relatively consistent within each group.

Figure 3 shows that typically four synergies were required to reconstruct both global and individual muscle activation for the stroke groups with mild or moderate impairment, similar to the number for the control and severely impaired stroke groups. More specifically, 4.0 ± 0.5 (3.8 ± 0.9) and 3.9 ± 0.6 (3.8 ± 0.9) synergies were identified from the stroke groups with mild and moderate impairment, respectively; similarly, 4.5 ± 0.7 (4.3 ± 0.9) synergies and 4.4 ± 0.5 (4.1 ± 0.6) synergies were identified from datasets of the control and severely impaired stroke groups (numbers out of parentheses determined by the MSE method; numbers within parentheses determined by VAF criteria (see Section Materials and Methods)). The number of synergies across groups was not significantly different (ANOVA, *F*_(3,32)_ = 2.24, *p* = 0.102, by the MSE method; ANOVA, *F*_(3,32)_ = 0.9, *p* = 0.45, by the VAF criteria). The global VAFs with four synergies were 94.6 ± 2.1% for the control group (mean ± SD; *n* = 12) and 96.1 ± 1.7, 95.9 ± 1.4, and 95.2 ± 1.1% for the stroke groups (*n* = 8) with mild, moderate, and severe impairment, respectively. All values were significantly greater than the chance level, 80.1–86.4% (*p* < 0.05). Some datasets required more or less than four synergies to meet our reconstruction criteria and the number was not sensitive to small changes in the criteria (see Section Materials and Methods). When more than four synergies were required, the additional synergies enabled fulfillment of our local criterion for individual muscles, rather than the global criterion. Since four synergies generally provided excellent EMG data reconstruction, we extracted four synergies from all datasets to facilitate comparison of synergies within and across groups. The distributions of muscle weights for the four synergies are summarized in Figure 4 across subjects in each group, with the group mean and standard deviation of muscle weights superimposed on each distribution.

Among the four synergies for subjects in each group, two involved mostly isolated activation of elbow flexors (BRD and BI) and elbow extensors (TRI_long_ and TRI_lat_), respectively (see Figure 4, elbow flexor (E Flex) and extensor (E Ext) synergies). As such, the elbow synergies of the mildly and moderately impaired stroke groups (Figures 4B,C) appeared similar to those observed for the control and severely impaired stroke groups. In controls, a third synergy, the “shoulder adductor/flexor (S Add/Flex)” synergy, was dominated by activation of BI, AD, MD, and PECT_clav_ (Figure 4A). In stroke survivors, as the impairment level increased from mild to severe, the S Add/Flex synergy tended to have activation of PECT_clav_, with a marginal activation of AD and MD (Figures 4B–D). The remaining “shoulder abductor/extensor (S Abd/Ext)” synergy in controls typically involved activation of MD and PD with one or more elbow muscles (Figure 4A). In contrast, in stroke survivors, the three heads of deltoid tended to be activated together within a single deltoid synergy as the impairment level increased (Figures 4B–D). These alterations in synergy structure are captured by the group mean and distribution of muscle weights shown in Figure 4.

**Figure 5:**
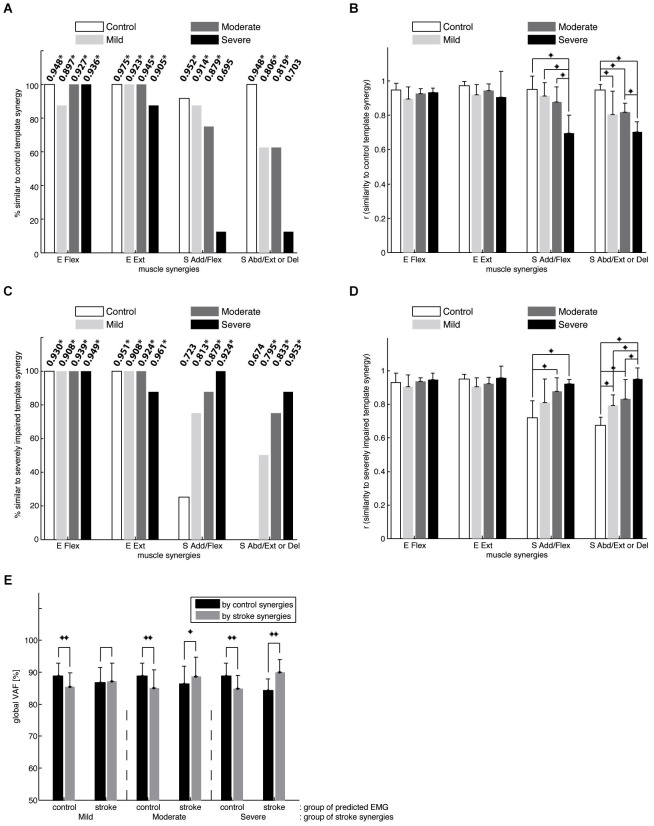
**Comparison of synergies in the control and stroke groups**. **(A)** the percentage of datasets in each group with a synergy similar to the mean synergies for the control group. The number at the top of each bar indicates the group mean of the *r*-values for each synergy (means that are significantly greater than chance level are denoted using asterisks; *p < 0.05). Note that elbow muscle synergies were typically conserved in the three stroke groups, while shoulder-related synergies were altered in individual stroke survivors, particularly those with severe impairment. **(B)** the average similarity of the mean control synergies to those observed for individual control and stroke subjects. In contrast to the elbow synergies, the average similarity for the shoulder synergies was group-dependent (* denotes a significant difference between group means; *p* < 0.05). **(C)** the percentage of datasets in the control and stroke groups, with a synergy similar to the mean synergies for the stroke group with severe impairment. **(D)** the average similarity of the mean severely-impaired group synergies to those observed for individual control and stroke subjects. **(E)** the goodness of reconstruction (VAFs) associated with cross-fitting synergies for subjects in the same group were significantly higher than those obtained by cross-fitting synergies for subjects in different groups (*p < 0.05; **p < 0.0001), implying intergroup differences in synergy structure. While black bars indicate the global VAFs obtained by fitting control synergies, gray bars represent those obtained by fitting synergies of one of the three stroke groups.

As described in Data Analysis, we compared synergies for each subject to synergy templates for the control and severely impaired stroke groups. These templates were effectively identical to the mean synergies shown in Figures 4A,D *(scalar product (r) greater than 0.99 in all cases)*. Figure 5A shows, for each synergy, the percentage of datasets in the control (*n* = 12) and stroke groups (*n* = 8 per group), with a synergy similar to the control template (i.e., r_norm greater than ~0.8, the typical threshold level). In addition, the group mean r_norm values for each synergy are indicated, with asterisks used to denote those that were significantly greater than chance level (*p* < 0.05). The percentage of synergies similar to the normative elbow synergies was not significantly different across groups (Fisher’s exact test, *p* > 0.6 for both E Flex and E Ext, respectively). This result is consistent with preservation of the elbow related synergies in chronic stroke survivors, irrespective of impairment level. In contrast, the percentage was group dependent for the S Add/Flex and S Abd/Ext (or Del) synergies (Fisher’s exact test, *p* < 0.01, respectively). *Post hoc* analyses showed that the percentages of altered S Add/Flex and S Abd/Ext synergies, respectively, were significantly different between the control and severely impaired stroke groups (*p* < 0.001). Other pair-wise comparisons across groups were not significantly different.

Figure 5B summarizes the results of ANOVA performed with r_norm for each muscle synergy. ANOVA confirmed a significant effect of group on r_norm for the two shoulder synergies (S Add/Flex, *F*_(3,32)_ = 14.15, *p* < 0.0001; S Abd/Ext or Del, *F*_(3,32)_ = 14.75, *p* < 0.0001; ^*^p < 0.05 for *post hoc* tests). Specifically, for the S Add/Flex synergy, r_norm differed between the severely impaired stroke group and each of the remaining groups. As shown in Figure 4, this result reflected the decrease in the muscle weights for AD and/or MD in severely impaired stroke survivors. In addition, for the S Abd/Ext or Del synergy, r_norm was different between the control and each of the three stroke groups and between the moderately and severely impaired groups (due to relatively large variance in the case of mild impairment, there was no significant difference between groups with mild and severe impairment). These results reflect the increase in the muscle weights for AD and MD across stroke groups and the absence of BRD in the synergy for stroke survivors. Overall, the results show that the structure of shoulder synergies was altered in mildly and moderately impaired stroke survivors, as well as the group with severe impairment. Figure 5C summarizes, for each group, the percentage of synergies similar to the template for the severely impaired stroke group (see Figure 4D). For the elbow synergies, the percentage was not group-dependent (Fisher’s exact test, *p*-value not available (due to 100% for all four groups) and *p* > 0.6 for E Flex and E Ext, respectively). However, the percentage was significantly different for S Add/Flex and S Abd/Ext (or Del) synergies (Fisher’s exact test, *p* < 0.001, respectively; Figure 5C). *Post hoc* analyses demonstrated that the percentage for the S Add/Flex synergy of the age-matched control group was significantly different from that of mildly and moderately impaired stroke groups, as well as the severely impaired group (*p* = 0.004, 0.001, and *p* < 0.001, respectively). Similarly, the percentage for the S Abd/Ext synergy of the control group was significantly different from that of the mildly, moderately, and severely impaired stroke groups (*p* = 0.001, 0.001, and *p* < 0.001, respectively). Figure 5D shows that ANOVA confirmed a significant effect of group on r_severe for the two shoulder synergies (S Add/Flex, *F*_(3,32)_ = 7.68, *p* < 0.001; S Abd/Ext or Del, *F*_(3,32)_ = 15.72, *p* < 0.0001; ^*^p < 0.05 for *post hoc* tests). Again, the results confirm that the altered structure of shoulder synergies in stroke survivors with severe impairment is also present in mildly and moderately impaired stroke survivors.

Muscle synergies considered as a set were also significantly different between the control and stroke groups (Figure 5E). Mean within-group global VAFs, determined by fitting each subject’s four synergies to the EMGs of the remaining subjects within the same group, were 88.8 ± 4.0 % for the control group and 87.2 ± 5.7, 88.6 ± 6.1, and 89.9 ± 4.1 % for the three stroke groups (mildly, moderately, and severely impaired), respectively. In comparison, across-group global VAFs, obtained by fitting synergies of each subject in a group to the EMGs of a subject in a different group, were significantly smaller (*t*-tests, *p* < 0.0001). For example, muscle synergies of control subjects were better able to predict the EMGs of the control group (the 1st black bar) than the same number of synergies of the mildly impaired group (the 1st gray bar). Similar trends were evident for muscle VAF (not shown as a figure). Consistent with intergroup differences in the structure of individual synergies, control synergies were better able to reconstruct the activation of elbow muscles (*t*-test, *p* < 0.0001; VAF of elbow muscles 87.2 ± 11.1%), compared with shoulder muscles (83.7 ± 13.2 %), of the affected limb, averaged across all stroke groups.

### Cluster analysis of muscle synergies

The results of Figure 5 show that, while the structure of muscle synergies in the control and severely impaired stroke groups was rather homogeneous, synergy structure for the mildly and moderately impaired stroke groups was more variable. Thus, we pooled the four muscle synergies for each stroke survivor and each limb of control subjects and grouped them using cluster analysis. This analysis enabled us to understand how four synergies of each group (the control and three stroke groups) could be classified.

**Figure 6:**
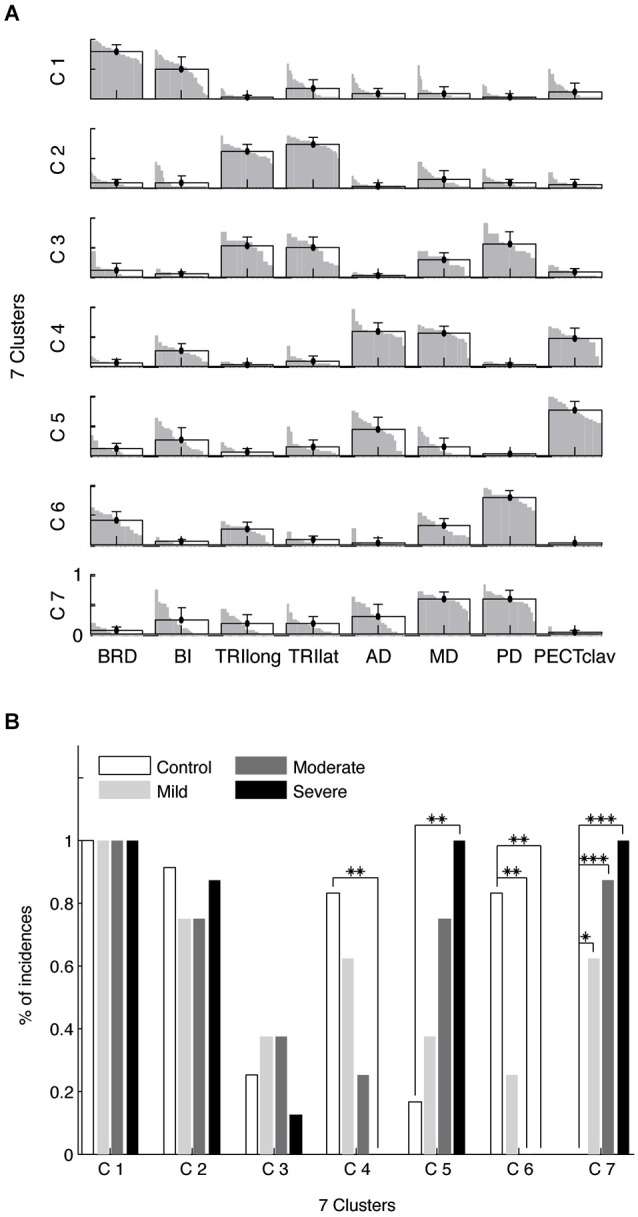
**Composition (A) and incidence (B) of muscle synergy clusters. Four synergies were identified for each limb and clustered into seven groups (C1-7)**. For each cluster, the group mean muscle weights and standard deviations are superimposed on the distribution of the corresponding muscle weights. Two clusters, C1-2, involved two elbow synergies, corresponding to E Flex and E Ext in Figure 4. C3 includes some E Ext synergies with activation of elbow extensors as well as shoulder abductor muscles. The S Add/Flex synergy tended to be classified into two clusters (C4-5). As the level of motor impairment increased, S Add/Flex synergies in Figure 4 tended to be more frequently categorized into C5, but less frequently into C4 **(B)**. Similarly, as the severity in motor deficit increased, S Abd/Ext synergies in Figure 4 were more frequently classified into C7, but less often into C6 **(B)**. The incidences of abnormal shoulder muscle synergies vary with the level of motor impairment following stroke (*, *p* = 0.004; **, *p* < 0.001; ***, *p* < 0.0001).

Figure 6A illustrates the seven identified synergy clusters of synergies (C1-7) as the mean and standard deviation of muscle weights superimposed on the distribution of muscle weights for the included subjects. Figure 6B summarizes the incidence of each synergy across groups. Clusters C1-C3 were comprised predominately of the E Flex and E Ext synergies identified in Figure 4. With reference to Figure 6B, the first two clusters (C1-2) were present in the vast majority of subjects and their incidence did not differ across groups (Fisher’s exact test, *p*-value not available (due to comparing four 100% incidences) and *p* > 0.6, respectively). Cluster 3 (C3) was comprised of synergies indicated as either E Ext or S Abd/Ext synergies in Figure 4, with co-activation of elbow extensors and shoulder abductors. The incidence of C3 did not vary with group (Fisher’s exact test, *p* > 0.6). These results suggest that elbow synergies are not altered following stroke, regardless of motor impairment level.

In contrast, the incidence of shoulder-related clusters (C4-C7) varied with the level of motor impairment. All synergies grouped as clusters 4 and 5 (C4 and C5) were S Add/Flex synergies, while all synergies in clusters 6 and 7 (C6 and C7) were S Abd/Ext synergies in Figure 4. For example, 25% and 75% of S Add/Flex synergies in moderate stroke were classified into C4 and C5, respectively. Note that across the stroke groups the incidences of C4 and C6, containing the shoulder synergies for the vast majority of control arms, decreased systematically with impairment level (Fisher’s exact test, *p* < 0.01 and *p* < 0.001, respectively). Conversely, the incidences of C5 and C7, which were identified almost exclusively in stroke survivors, increased with impairment severity (Fisher’s exact test, *p* < 0.01 and *p* < 0.0001, respectively). *Post hoc* analyses (see Figure 6B) confirmed these trends.

### Potential impact of inter-group imbalances

With reference to Table 1, ANOVA indicated that the stroke groups were imbalanced with respect to time since stroke-onset (*p* < 0.01) and Modified Ashworth score (*p* < 0.0001 and *p* = 0.19 for elbow flexion and extension, respectively). For each muscle synergy, multiple regression was used to predict r_norm (*n* = 24) with FM subscore, Modified Ashworth score for elbow flexion, and time after stroke-onset as independent variables. Modified Ashworth for elbow extension was not correlated with the r_norm of any synergies (*p* > 0.05), and therefore was not considered as an independent variable in the model.

Initial modeling including all three independent variables revealed that only FM subscore was a significant predictor of r_norm, and then only for the shoulder synergies. Variance inflation factors (VIFs) were <3 for each independent variable. Subsequent linear regressions confirmed that FM subscore was a significant predictor of r_norm for both the S Add/Flex synergy (*F*_(1,22)_ = 14.095, *p* < 0.01, *R*^2^ = 0.390) and S Abd/Ext synergy (*F*_(1,22)_ = 5.251, *p* < 0.05, *R*^2^ = 0.193).

## Discussion

In this study, we quantified how stroke impacted the structure of muscle synergies underlying isometric force generation in the upper extremity of stroke survivors with mild and moderate impairment, as compared to the age-matched control and stroke survivors with severe impairment. We found that a modular organization of force generation was preserved for mild and moderate as well as severe impairment levels. In the post-stroke individuals, four muscle synergies typically accounted for greater than 90% of the total variance of the EMG patterns collected from elbow and shoulder muscles, which was also the case for the age-matched control and severely impaired stroke groups. Of the four synergies identified, synergies with relatively isolated activation of the elbow flexors and extensors were conserved following stroke, irrespective of impairment level. However, the two synergies dominated by activation of shoulder muscles were altered in some stroke survivors with mild and moderate impairment, as observed in most of our post-stroke individuals with severe impairment. The alterations in the structures of the shoulder synergies may reflect an impaired ability to differentially activate the three heads of deltoid. Cluster analysis showed that the incidences of impaired and unimpaired shoulder synergy patterns were positively and negatively correlated with impairment level, respectively. Overall, our results indicate that alterations in proximal muscle synergy structure can appear in mild and moderate stroke, but most distinctly in severely impaired hemiparetic individuals.

### Mechanisms of abnormal post-stroke muscle coordination in various motor behaviors

Recent studies have consistently reported that after stroke, there is preservation of a low-dimensional modular organization of muscle coordination in both the human upper (Cheung et al., 2009b, 2012; Roh et al., 2013) and lower (Clark et al., 2010) extremities, as well as the hand (Lee et al., 2013).Initial studies of post-stroke muscle activation patterns attributed coordination disturbances to abnormal activation (or recruitment) of preserved muscle synergies. For example, Clark et al. (2010) showed that the structure of the muscle synergies (i.e., the muscle weights within a muscle synergy) identified during walking was similar between healthy and post-stroke groups. However, an impaired ability to independently recruit intact muscle synergies seemed to reduce locomotor output complexity in severely impaired individuals. Similarly, in cases of severe impairment (FM < 30), fewer synergies were required to explain the variance of EMGs recorded from the affected limb during reaching, reflecting a merging of multiple normal muscle synergies (Cheung et al., 2012). These results are consistent with the view that synergy structure may be mainly specified at lower neural centers (e.g., spinal cord and brainstem) (Roh et al., 2011), while synergy activation may be controlled by higher centers that are likely to be directly impacted by stroke (Lee et al., 2013; Roh et al., 2013). However, this view does not preclude any potential contribution of higher neural divisions (ex. a cortical influence) to synergy expression.

In contrast, recent studies have reported post-stroke alterations in the composition of synergies underlying muscle activation during isometric tasks (Cruz and Dhaher, 2009; Lee et al., 2013; Roh et al., 2013). Lee et al. found that post-stroke muscle synergies involving hand and wrist muscles could not be well represented as a simple merging of muscle synergies recorded in healthy subjects, indicating a significant change in muscle synergy structure. More interestingly, studies of both the hand and the upper extremity show greater coactivation among muscles in stroke survivors (e.g., a higher correlation of extensor digitorumcommunis-extensor carpi radialis and first dorsal interosseous-extensor digitorumcommunis at the hand and coactivation of the three heads of deltoid in the arm), especially in stroke subjects with severe impairment. We reason that a lack of individuated muscle control available in the healthy state can be an underlying mechanism of alterations in the structure of muscle synergies in the human hand and arm.

Among the muscle synergies identified during our static force generation protocol, the synergies underlying the activations of proximal muscles were altered post-stroke. As compared to the S Add/Flex synergy for control subjects, the activations of the anterior and medial heads of deltoid were dissociated from activation of the clavicular fibers of pectoralis major. Instead, the two heads of deltoid appeared to combine with the activation of the posterior deltoid to form a general post-stroke deltoid synergy.

Inappropriate coactivation of agonist and antagonist muscles is a frequently reported consequence of stroke (Knutsson and Richards, 1979; Conrad et al., 1985; Hammond et al., 1988; Dewald et al., 1995; Levin and Dimov, 1997; Reinkensmeyer et al., 2002). For example, reaching movements performed with the paretic arm exhibit a higher level of co-activation of anterior and posterior heads of deltoid across different load types, as compared to controls without neurologic involvement (Stoeckmann et al., 2009). Similarly, increased activation of the lateral deltoid, in addition to the anterior deltoid, was observed during reaching movements of the affected arm (McCrea et al., 2005). McCrea et al. concluded that the coactivation was associated with a compensatory response to weakness of the anterior deltoid, which exhibited saturation of activation. In contrast, our study involved relatively low force levels, and muscle activation was well below maximum. Accordingly, we reason that post-stroke disruption of the ability to dissociate activation of the deltoid heads may be an alternative mechanism that explains the co-activation of shoulder muscles during reaching.

### Study limitations

A limitation of this study is that there were imbalances between stroke groups in the level of spasticity and time since stroke-onset. Multiple regression analyses indicated that the similarity of post-stroke S Add/Flex and S Abd/Ext synergies to normative control synergies depended only on the FM subscore, and not on time since onset or Modified Ashworth score. Variance inflation factors for all variables were less than 3, reflecting a relatively moderate level of correlation among predictor variables. However, the influence of time since stroke-onset merits further study, particularly since an earlier study (Cheung et al., 2012) reported that post-stroke alterations in muscle synergies were correlated with time after stroke-onset. Conceivably, the alterations in shoulder synergies reported in the current study arise gradually over time, potentially in association with the compensatory response to shoulder weakness described by McCrea et al. (2005).

### The usefulness of the isometric protocol to dissociate the motor performance in control and stroke groups

To examine the impact of stroke on modular motor control, we focused on isometric upper extremity force tasks. While intergroup differences in task performance (e.g., kinematics) may confound interpretation of results in dynamic tasks, the isometric protocol provided an opportunity to more closely match task variables across healthy and hemiparetic individuals. The relative activations of muscles depend on joint angle and velocity (Nakazawa et al., 1993; Leedham and Dowling, 1995; Hwang and Abraham, 2001), parameters that could vary across subjects during dynamic tasks, especially hemiparetic individuals with differing levels of impairment. Our isometric protocol also provides better control of the potential impact of weakness on task performance. We reason that the general similarity of muscle synergies observed across subjects within each group (Figure 4) reflected, in part, better control of biomechanical factors during task performance.

Previous studies of the modular control of post-stroke movements have reported a reduced number of muscle synergies, correlated with the level of motor impairment (Bowden et al., 2010; Clark et al., 2010). However, we did not find a similar reduction in the number of synergies underlying isometric force generation in our stroke survivors. Since there is no mathematical formula to identify the number of muscle synergies, researchers have used several different methods to estimate the appropriate number of synergies (Cheung et al., 2005; Clark et al., 2010; Torres-Oviedo and Ting, 2010). To objectively perform the identification of the synergy number, we applied two methods that resulted in approximately four synergies across control and stroke groups. 3-D force generation across the full range of directions requires at least four synergies (Leijnse et al., 2005), consistent with the results of our analysis. Thus, the preservation of muscle synergy number across groups in our study may be associated with this task constraint.

### Implications for neurorehabilitation

Our study shows that alterations in muscle synergies are evident in stroke survivors with mild and moderate impairment, and the incidence of impaired synergies is positively associated with clinical impairment level. Similarly, the studies of human reaching (Cheung et al., 2012), locomotion (Bowden et al., 2010; Clark et al., 2010) and isometric hand tasks (Lee et al., 2013) show alterations in muscle synergies or their activations most prominently in severely impaired stroke survivors, and to a lesser extent in subjects with mild-to-moderate impairment. Greater changes in muscle synergies or their activations correspond to an overall reduction in complexity of EMG responses or motor control, and this reduction in output complexity tends to be associated with poorer motor performance (Bowden et al., 2010; Clark et al., 2010). Thus, muscle synergy analysis appears to be useful to identify abnormalities in muscle coordination in terms of an altered number, structure, and activation of muscle synergies (Clark et al., 2010; Cheung et al., 2012; Lee et al., 2013; Roh et al., 2013). The qualitative differences in the nature of the synergy abnormalities across impairment levels highlight the need for development of subject-specific rehabilitation approaches.

Recent studies show that targeted motor training can modify kinematic synergies (Dipietro et al., 2007) and abnormal isometric torque patterns (Ellis et al., 2005). These results support the possibility to develop novel therapeutic protocols targeting restoration of normal muscle synergy structure and recruitment. For example, in stroke survivors with motor disturbances related to abnormal recruitment of normal muscle synergies, one potential approach is to provide visual or proprioceptive biofeedback based on the difference between the healthy and abnormal synergy recruitment. Specifically, feedback directly related to the dysfunctional synergy activation coefficients can assist stroke patients in generating normal muscle activation patterns, which would facilitate task performance during training (d’Avella et al., 2013). The design of therapies to normalize synergy structure seems more problematic. However, muscle synergy analysis can guide a subject-specific functional electrical stimulation (FES) approach to identify which muscle groups to stimulate to promote a desired synergy structure (Piazza et al., 2012). Thus, muscle synergy analysis can provide a framework for characterizing post-stroke motor impairments, designing clinical treatments, and tracking rehabilitation progress.

Determination of the degree to which muscle synergies are similar across different motor behaviors (e.g., static vs. dynamic motor tasks) may provide insight into the potential generalization of functional gains to non-trained tasks. In healthy individuals, the synergies underlying isometric force generation (Roh et al., 2013) and reaching movements (Cheung et al., 2009b) appear comparable, suggesting that some synergies may be utilized across different motor tasks. Further studies on the generalization of muscle synergies, both in healthy individuals and stroke survivors (Cruz and Dhaher, 2009), are warranted. This may provide a theoretical foundation for the development of well-controlled isometric training protocols as therapeutic interventions for individuals with stroke.

## Author contributions

Author contributions: Jinsook Roh, William Z. Rymer, and Randall F. Beer performed conception and design of research; Jinsook Roh performed experiments; Jinsook Roh analyzed data; Jinsook Roh, William Z. Rymer, and Randall F. Beer interpreted results of experiments; Jinsook Roh prepared figures; Jinsook Roh drafted manuscript; Jinsook Roh, William Z. Rymer, and Randall F. Beer edited and revised manuscript; Jinsook Roh, William Z. Rymer, and Randall F. Beer approved final version of manuscript and agreed to be accountable for all aspects of the work.

## Conflict of interest statement

The authors declare that the research was conducted in the absence of any commercial or financial relationships that could be construed as a potential conflict of interest.
